# The Timing of Melatonin Administration Is Crucial for Its Antidepressant-Like Effect in Mice

**DOI:** 10.3390/ijms19082278

**Published:** 2018-08-03

**Authors:** Rosa Estrada-Reyes, Marcela Valdés-Tovar, Daniel Arrieta-Baez, Ana María Dorantes-Barrón, Daniel Quero-Chávez, Héctor Solís-Chagoyán, Jesús Argueta, Margarita L. Dubocovich, Gloria Benítez-King

**Affiliations:** 1 Laboratorio de Fitofarmacología, Dirección de Investigaciones en Neurociencias, Instituto Nacional de Psiquiatría Ramón de la Fuente Muñiz, Calzada México-Xochimilco 101, San Lorenzo Huipulco, Tlalpan, Ciudad de México 14370, Mexico; restrada@imp.edu.mx (R.E.-R.); ana1967@imp.edu.mx (A.M.D.-B.); 2 Laboratorio de Neurofarmacología, Subdirección de Investigaciones Clínicas, Instituto Nacional de Psiquiatría Ramón de la Fuente Muñiz, Calzada México-Xochimilco 101, San Lorenzo Huipulco, Tlalpan, Ciudad de México 14370, Mexico; mvaldes@imp.edu.mx (M.V.-T.); quero.rude.daniel@gmail.com (D.Q.-C.); hecsolch@imp.edu.mx (H.S.-C.); jadclear@gmail.com (J.A.); 3 Instituto Politécnico Nacional CNMN Luis Enrique Erro s/n Unidad Profesional Adolfo López Mateos, Gustavo A. Madero, Ciudad de México 07738, Mexico; darrieta@ipn.mx; 4 Department of Pharmacology and Toxicology, Jacobs School of Medicine and Biomedical Sciences, University at Buffalo (SUNY), 955 Main Street, Buffalo, NY 14203, USA; mdubo@buffalo.edu

**Keywords:** melatonin, zeitgeber time, antidepressant-like effect, behavioral despair, forced-swimming test, tail suspension test

## Abstract

Melatonin is synthesized by the pineal gland with a circadian rhythm in synchrony with the environmental light/dark cycle. A gradual increase in circulating levels of melatonin occur after lights off, reaching its maximum around the middle of the dark phase. Agonists of melatonin receptors have proved effectiveness as antidepressants in clinical trials. However, there is contradictory evidence about the potential antidepressant effect of melatonin itself. Herein we studied melatonin administration in mice at two zeitgeber times (ZT; ZT = 0 lights on; 12:12 L/D), one hour before the beginning (ZT11) and at the middle (ZT18) of the dark phase after either a single or a three-dose protocol. Behavioral despair was assessed through a forced-swimming test (FST) or a tail suspension test (TST), at ZT18.5. A single dose of 4 mg/kg melatonin at ZT11 was effective to reduce the immobility time in both tests. However, acute administration of melatonin at ZT18 was not effective in mice subjected to FST, and a higher dose (16 mg/kg) was required to reduce immobility time in the TST. A three-dose administration protocol of 16 mg/kg melatonin (ZT18, ZT11, and ZT18) significantly reduced immobility time in FST. Data indicate that the timely administration of melatonin could improve its antidepressant-like effect.

## 1. Introduction

In vertebrates, melatonin (*N*-acetyl-5-methoxytryptamine) is synthesized by the pineal gland during the dark phase of the light/dark cycle. This gland receives a neuronal noradrenergic input from an efferent pathway originating in the suprachiasmatic nucleus. In response to this signal, pinealocytes synthesize melatonin [1]. This indolamine is released into the cerebrospinal fluid (CSF) and the general circulation [2,3]. Thus, circulating melatonin levels oscillate following a circadian rhythm reaching the maximum amplitude during the dark phase of the environmental illumination cycle [1]. Melatonin reaches the CSF directly by the pineal recess [4,5] and from this fluid it is distributed into different brain regions providing the photoperiodic information to the neurons. The nocturnal concentration of melatonin in the CSF is 100-fold higher than in plasma [6]. Recently it was described that the brain tissue is bathed by the melatonin accumulated in the CSF and not by the blood circulating melatonin [6]. In the brain, melatonin is also synthesized in extrapineal sites such as the cerebellum and cortex [7,8]. However, current evidence indicates that melatonin synthesized in extrapineal sites does not contribute to the plasma circulating melatonin and is not related to the environmental photoperiod (for a review see Acuña-Castroviejo et al., 2014) [9].

Melatonin is transported from the CSF into the cells by passive diffusion and by the GLUT1 transporter and PEPT1/2 (for a review see Mayo et al., 2017) [10]. Once inside the cells melatonin acts either directly (e.g., as a free radical scavenger) or through its interaction with diverse intracellular targets such as calmodulin, protein kinase C (PKC) and some members from the retinoid-related orphan receptor family. Melatonin also exerts many of its actions through the activation of MT1 and MT2 G-protein coupled receptors [11].

In the central nervous system (CNS) as in the rest of the organism, melatonin has pleiotropic modulatory effects and acts as a neuroprotective substance through its antioxidant properties [11,12]. Additionally, this indolamine prompts neurodevelopment, is an anti-apoptotic molecule and modulates neuroinflammation among other actions [13,14,15,16,17]. The hippocampus, the brain region which integrates spatial memory, learning and cognition [18,19], is also one of the main structures affected in patients with major depression, showing atrophy and a decreased volume size by brain imaging studies [20]. This brain structure has also been extensively studied as a target for melatonin effects in rodent models. These studies have shown that melatonin protects hippocampus from oxidative stress [21] and induces neuroplasticity at different levels, from neurogenesis and cell survival in the dentate gyrus [22,23], as well as axonogenesis in hippocampal neural precursors [24], to dendritogenesis and synaptic cluster formation in hilar neurons [25,26]. On the other hand, it has been shown that melatonin improves cognition and modulates behavioral despair and other anhedonic- and anxiety-like behaviors in rodent models [27,28,29,30], and when the indolamine is administered together with selective serotonin reuptake inhibitors (SSRIs) it synergizes the antidepressant-like effects of these compounds in the forced swimming test (FST) [31,32]. Despite this information, contradictory evidence exists regarding the appropriate protocol and route of administration of melatonin necessary to induce antidepressant-like effects. For instance, melatonin is either administered orally in drinking water, intraperitoneally (i.p.) or intracerebrovascularly (i.c.v.) [23,30,33]. Additionally, the time of administration with respect to the light/dark cycle (zeitgeber time (ZT); ZT = 0 lights on), the doses, and the rodent species vary in each study reported. Thus, acute administration of melatonin decreased the immobility time in the tail suspension test (TST) performed between ZT4 and ZT9 when administered either i.p. (0.1–30 mg/kg) or i.c.v. (0.01–1 mg/kg) [30].

In contrast, acute administration of melatonin (2.5–10 mg/kg, i.p.) failed to induce an antidepressant-like effect in mice subjected to the FST. Melatonin administered at 0.01–1 mg/kg doses 30 min before the FST performed at early dark or middle of night did not change the immobility time in comparison with vehicle in rodents. However, daily administration of pharmacological doses of melatonin significantly reversed the increase in the immobility time observed in a rodent model of depression induced by chronic exposure to the FST [34]. Thus, herein we studied two different melatonin administration protocols at key zeitgeber times according to an endogenous melatonin secretion profile, i.e., a single acute i.p. dose of melatonin at either ZT11 or ZT18, or a three-dose protocol at ZT18, ZT11, and ZT18. These times correspond to one hour before dark phase begins (ZT11) and to the middle of the dark phase (ZT18). Behavioral tests were assessed at ZT18.5 in all cases. Data showed that a single melatonin dose (4 mg/kg) reduced the immobility time in mice subjected to either FST or TST, when administered at ZT11. In contrast, acute administration of the indolamine at ZT18 was only effective in reducing immobility time in the TST at a higher dose (16 mg/kg). However, a robust antidepressant-like effect compared with fluoxetine (FLX) was observed in the FST when melatonin was administered in a three-dose protocol. Data indicate that timing administration is critical for melatonin’s antidepressant-like effect in mice.

## 2. Results

### 2.1. Nocturnal Circulating Melatonin in Swiss Webster Mice

**Figure 1:**
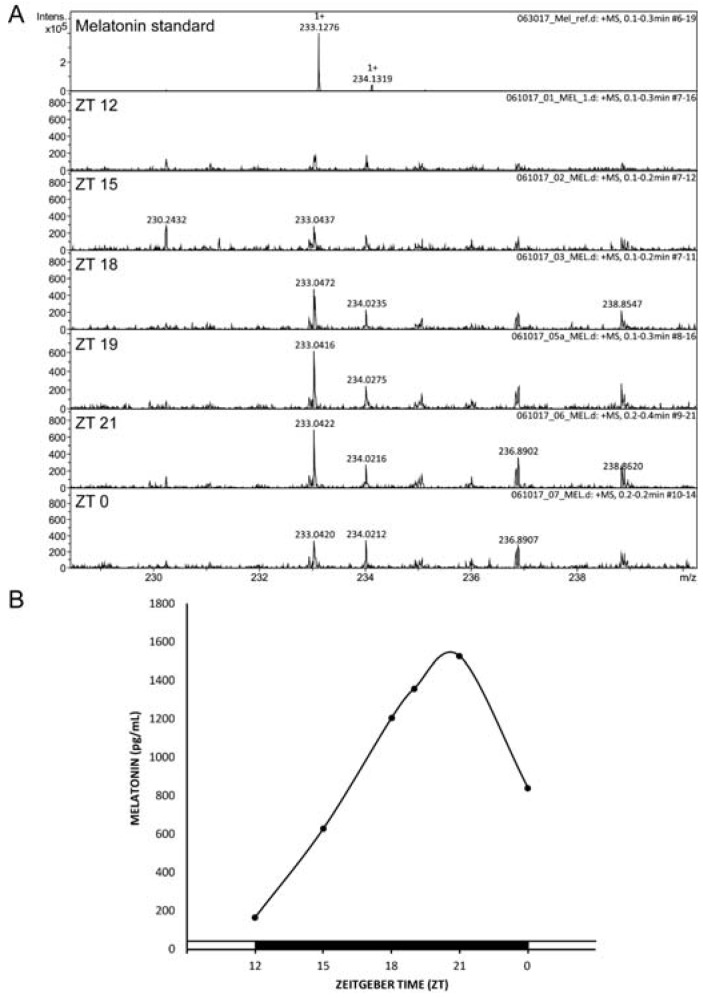
Swiss Webster mice serum melatonin levels through the dark phase. (**A**) DIESI/MS mass spectra diagrams of melatonin at different zeitgeber times (ZT). (**B**) Melatonin levels at ZT12, ZT15, ZT18, ZT19, ZT21, and ZT0. Data are derived from one mouse for each ZT.

The circadian production of melatonin has been studied in serum and CSF in several vertebrate species, including rodents [1]. The Swiss Webster mice strain has been used to evaluate melatonin antidepressant-like effects [30,34]; however, it is not clear if melatonin is synthesized in the retina and/or in the pineal gland of this mice strain [35,36]. Circulating melatonin levels in the Swiss Webster mice strain were detected in non-extracted serum samples obtained during the dark phase where levels of the indolamines are expected to be high using the Direct Infusion Electrospray Ionization Mass Spectrometry (DIESI/MS) analysis. Samples were obtained every 3 h from ZT12 (time when lights off), as well as at ZT19, considering that some mice strains display a very sharp peak of circulating melatonin at that precise time [37]. Figure 1A shows a peak of [M^+1^] 233 uam (units of atomic mass) detected in the serum samples coinciding with the melatonin standard. A gradual increase in melatonin levels was observed after lights off with a maximum between ZT19 and ZT21 (Figure 1B), suggesting that the Swiss Webster mice synthesize melatonin during the dark phase. In our study, we used a method with high selectivity and efficiency allowing direct detection of melatonin without extraction and overcoming the high nonspecific binding of melatonin to serum bovine albumin and hemoglobin present in hemolyzed samples, which leads to low reproducibility and aberrant results [38].

### 2.2. Melatonin-Mediated Antidepressant-Like Effect When Administered at ZT11

**Figure 2:**
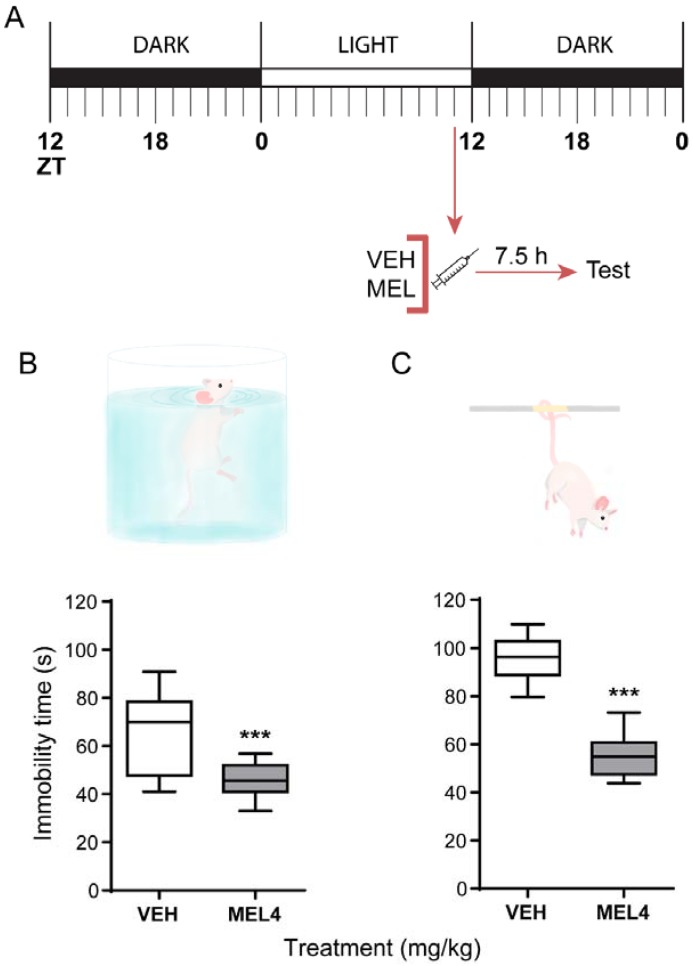
Effect of a single dose of melatonin at ZT11 on two behavioral tests in mice: forced swimming test (FST) and tail suspension test (TST). (**A**) Melatonin treatment protocol. (**B**) Effect of melatonin (4 mg/kg; MEL4) on the immobility time of mice subjected to the FST. (**C**) Effect of melatonin (4 mg/kg; MEL4) on immobility time in the TST. For each test, a vehicle-treated group (VEH) was included. Data show median and distribution for 8 mice per group. Statistical differences were assessed with Student *t*-test; *** *p* ≤ 0.001.

Melatonin was administered at ZT11, and behavioral tests were assessed 7.5 h later, around the middle of the dark phase when mice have their highest spontaneous physical activity (Figure 2A). Our results showed a significant reduction in the immobility time in mice treated with melatonin in both FST and TST (Figure 2B,C, respectively). It should be noted that, in the FST, melatonin induces antidepressant-like effect and clearly reduces the data dispersion (*t* = 2.486, df = 4, *p* = 0.013). These results indicate that a single dose of 4 mg/kg melatonin decreases the immobility behavior to 50 s for 76% of the experimental subjects (i.e., the power of one-tail *t*-test with α = 0.050 was 0.764).

On the other hand, a stronger response was observed in TST in comparison with the FST. Melatonin induced a significant reduction in the immobility behavior regarding the control group (F_(2,24)_ = 44.12, *p* ≤ 0.001) (Figure 2C). The magnitude of the response to melatonin in the TST was comparable to the one induced by the SSRI fluoxetine, a positive control for antidepressant-like behavioral tests.

Evidence indicates that, when exogenous melatonin is administered one hour before the endogenous indolamine starts to rise, we observed a robust antidepressant-like effect.

### 2.3. Acute Melatonin Administration at the Middle of the Dark Phase Reduced Immobility Only in the TST

**Figure 3:**
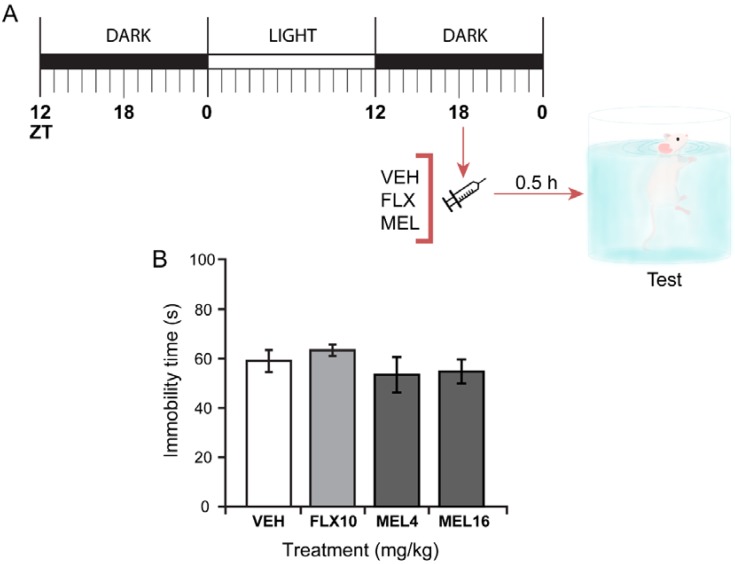
Single melatonin dose at ZT18 on mice forced swimming test (FST). (**A**) Melatonin treatment protocol. (**B**) Immobility time of mice subjected to FST following administration with vehicle (VEH), fluoxetine 10 mg/kg (FLX10), melatonin 4 mg/kg (MEL4) or 16 mg/kg (MEL16). Data represent mean and standard error of the mean for 8 mice per condition. Differences were assessed using a one-way ANOVA. *p* ≤ 0.05 for a significant difference.

We next compared the effect of single acute doses of melatonin (4 or 16 mg/kg) in the Swiss Webster mice FST to FLX (10 mg/kg), a SSRI that produces antidepressant-like effect in mice in the FST under a three-dose protocol but not after acute administration at ZT18 [39]. Neither melatonin nor FLX affected behavioral immobility 30 min after administration (Figure 3).

**Figure 4:**
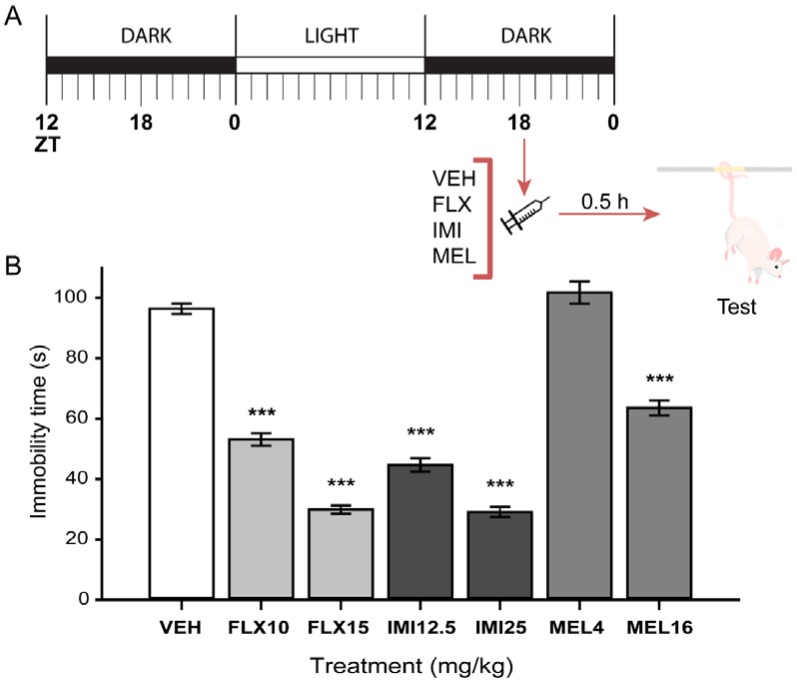
Single melatonin dose at ZT 18, on mice tail suspension test (TST). (**A**) Melatonin treatment protocol. (**B**) Immobility time of mice in TST. Treatments: Fluoxetine 10 mg/kg (FLX10) and 15 mg/kg (FLX15); imipramine 12.5 mg/kg (IMI12.5) and 25 mg/kg (IMI25); melatonin 4 mg/kg (MEL4) and 16 mg/kg (MEL16). Data represent mean and standard error of the mean for 8 mice per condition. Differences between groups were assessed with a one-way ANOVA followed by Bonferroni’s post-hoc test. *** *p* ≤ 0.001 when compared with vehicle (VEH) group.

The acute effect of MEL was also tested in the TST in mice at ZT18. As shown in Figure 4, MEL at 16 mg/kg but not at 4 mg/kg significantly decreased immobility time in the TST in comparison with the vehicle-treated group. Both FLX (10 and 15 mg/kg) and the tricyclic antidepressant imipramine (IMI; 12.5 and 25 mg/kg) reduced the time spent on immobility behavior (F_(6,49)_ = 129.44, *p* ≤ 0.001) as previously reported.

### 2.4. Three Melatonin Doses over a 24 Hour-Period Induced a Significant Antidepressant-Like Effect in the FST

**Figure 5:**
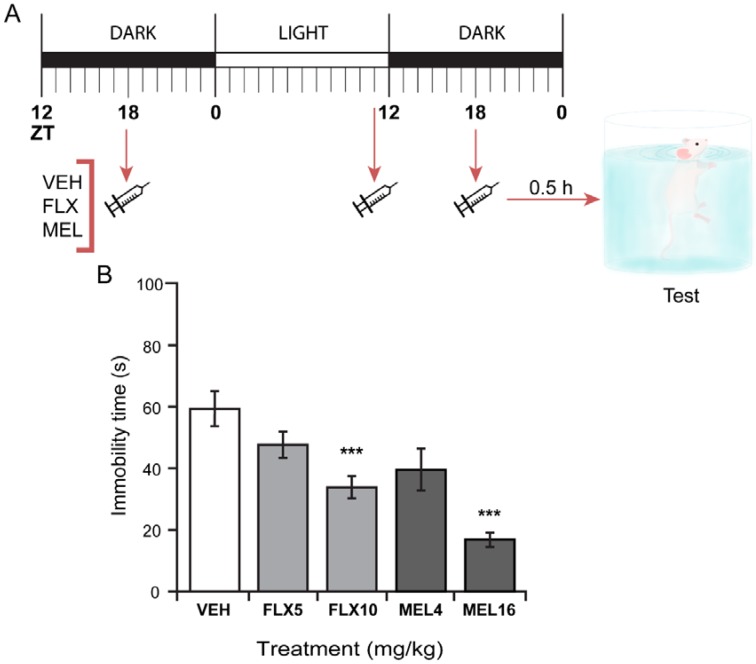
Effect of three doses of melatonin over a 24 h-period in the mice forced swimming test (FST). (**A**) Melatonin treatment protocol with three doses administrated at ZT18, ZT11, and ZT18. Mice were treated with either vehicle (VEH), 5 or 10 mg/kg fluoxetine (FLX5 and FLX10, respectively), or 4 or 16 mg/kg melatonin (MEL4 and MEL16, respectively). (**B**) Data shows mean and standard error of the mean for 8 mice per group. Statistically significant differences were assessed using a one-way ANOVA, followed by Bonferroni’s post-hoc test. *** *p* ≤ 0.001 when compared with the VEH-treated group.

Previously it was shown that SSRIs cause an effective antidepressant-like effect in mice tested in the FST when they were treated with a three-dose protocol [39]. In this work, we administered the first dose of melatonin at ZT18, the second at ZT11 of the next circadian cycle, and the third at ZT18, just 30 min before the behavioral test. In this protocol, exogenous melatonin would further increase the endogenous melatonin levels which are expected to be high at ZT18, and to anticipate its gradual raise at ZT11. Melatonin at 16 mg/kg administered using this three-dose protocol significantly decreased immobility time by up to 70% (Figure 5) (F_(2,31)_ = 11.57, df = 2, *p* ≤ 0.001). Melatonin at 4 mg/kg had no significant effect. The robustness of this response is even greater than that of FLX that induced a 44% decreased immobility in this protocol. These results suggest that a timely schedule of melatonin administration might induce a response as an antidepressant-like intervention, similar to that of clinically used antidepressants.

### 2.5. Melatonin Administration Does Not Alter General Locomotor Activity in Mice

Finally, to exclude an effect on immobility due to unspecific side-effects of melatonin on locomotor activity (e.g., hyper or hypoactivity), mice ambulatory activity was evaluated in the Open Field Test (OFT). Our results showed that ambulation levels of animals treated with melatonin were similar to those observed in the vehicle group, excluding a potential effect on swimming performance due to motor activity alterations by melatonin (Table 1). This data indicates that the effects of melatonin on immobility behavior are not due to an increase in ambulatory activity.

## 3. Discussion

Here we demonstrated that melatonin administered one hour before lights off (ZT11) decreased immobility in Swiss Webster mice when tested in the FST and in the TST, with this effect being more pronounced in the later test. Further, using a three-dose protocol administered at two key circadian times, melatonin induced an antidepressant-like effect in Swiss Webster mice. Serum melatonin was determined during the dark phase; however, previous work detected no melatonin secretion from the retina in this mice strain [35]. In that study, the detection of melatonin in Swiss Webster mice was done by radioimmunoassay in perfusates of the cultured media of retinas maintained ex vivo [35]. In the current study, we used a Direct Infusion Electrospray Ionization Mass Spectrometry (DIESI/MS) analysis method which has high sensitivity and selectivity. In Swiss Webster mice, melatonin is present in the serum at levels comparable to those found in other mice strains [40]. Serum melatonin levels slowly increase when lights are turned off at ZT12 reaching the maximal amplitude at ZT20, with levels decreasing after this time.

A dose of melatonin of 4 mg/kg in mice is comparable with pharmacological doses of 3–10 mg given to humans, taking into account the differences in metabolism between species [41,42]. Additionally, in previous studies in mice, the melatonin doses tested on behavioral paradigms were in the same range that we used here [30]. In addition, the effects of melatonin were tested in two different behavioral tests, the TST and FST. The TST is more sensitive than the FST [43], and both of them induce a passive behavior in mice to cope with an unescapable stressful situation. We showed that, in both paradigms, melatonin had a robust antidepressant-like effect after 7.5 h of its administration at ZT11. However, a stronger response was observed in the TST.

To further characterize the antidepressant-like effect of melatonin, we used the previously reported protocol for SSRI administration in the same mice strain employed here. These drugs do not produce an antidepressant-like behavior after an acute administration; however, administered following a three-dose protocol, they induced a robust antidepressant-like effect in mice [39]. Here, we demonstrated that acute administration of melatonin either at 4 or 16 mg/kg does not produce antidepressant-like effects in the FST when administered at ZT18 and tested 30 min later. In contrast, in the TST, which is less stressful than the FST and a more sensitive test, melatonin at the highest dose of 16 mg/kg induced a clear antidepressant-like effect similar to the caused by the positive reference drugs fluoxetine and imipramine. Further, both fluoxetine and melatonin following the three-dose protocol induced a robust antidepressant-like effect without altering the mice general ambulatory activity.

The mechanism by which melatonin causes an antidepressant effect has been partially characterized. MT1 receptor stimulation has been involved in this behavior [29,44]. In this regard, the density of melatonin receptors is highest towards the end of the light phase, when melatonin levels are low as demonstrated by the binding of [I^125^]-melatonin to receptors and by subcellular receptor localization studies [45,46,47]. Herein, we found that the antidepressant-like effect of melatonin was observed when administered 1 h before the endogenous melatonin circulating levels starts to increase. Given this evidence, and because melatonin receptors participate in this behavior, it is possible that, at this zeitgeber time, melatonin receptors may reached their maximal capacity to be stimulated. In contrast, we could not find the antidepressant-like effect when melatonin was administered at ZT18 and tested 30 min later, when melatonin receptors are partially occupied by endogenous melatonin [45,46,47]. One possible explanation could be that a lasting period of hours is necessary to observe the behavioral response, since it is known that the antidepressant behavior is related to brain plastic changes that take hours to be established (for a review see Valdés-Tovar et al., 2018) [48]. For instance, dendritic and axonal formation as well as synaptogenesis was reported to take place after 6 h of melatonin administration [13,25]. However, the changes observed under acute administration at ZT18 and tested 30 min later in the TST are also probably the result of electrophysiological fast responses as reported to be elicited by melatonin [30,49]. In contrast, the fact that melatonin administered at ZT18 and tested 30 min later in the FST had no effect could be explained because FST is a more stressful and less sensitive test than TST; the detection of the antidepressant-like effect of melatonin was therefore not possible in these conditions [50]. Further research would be required to disclose the precise melatonin-triggered signaling pathways and cellular events involved in modulating behavioral despair in mice.

Together, our results suggest that melatonin effectiveness is improved when its administration resembles its circadian profile since the three-dose protocol involves two zeitgeber times: when melatonin levels are low and when melatonin levels are high. To support this notion, it is important to mention that a daily administration of FLX in combination with melatonin attenuated the depressive-like behavior caused by a sub-chronic stress paradigm in mice [31]. Additionally, the serotonergic system has circadian variations [51]. For example, serotonin levels in the brain are increased during the night and its receptors during the day [52,53], supporting that the circadian administration of either SSRIs, melatonin or a combination of them, has an important role to elicit effective antidepressant actions. It is important to mention that SSRIs cause an increase of serotonin levels in the synaptic cleft [54]. In this regard, melatonin increases total serotonin levels in the brain [55]. Thus, the effects of both drugs on the serotonergic system could explain at least in part the synergic effects of SSRIs and melatonin in the antidepressant actions.

In conclusion, our overall results indicate that even a single pharmacological dose of melatonin may exert an antidepressant-like effect in mice if it is properly timed. This proper timing would depend on the cellular and biochemical underlying mechanisms of melatonin-mediated behavior modulation. In this work, we found that the indolamine may act at least in two fashions: (1) an acute minute-scale one that might involve rapid Ca^2+^-triggered events and/or synaptic spine remodeling and (2) an hour-scale one that may involve more complex events, such as the induction of dendritogenesis, synaptogenesis, and de novo protein synthesis. Interestingly, in the three-dose protocol, these two mechanisms of action might add to or synergize each other to produce a potent effect, comparable to well-known antidepressant drugs. Undoubtedly, further research is needed for an in-depth understanding of melatonin-mediated mechanisms involved in behavioral modulation.

## 4. Material and Methods

### 4.1. Animals

Male Swiss Webster mice (25–35 g) were obtained from the vivarium of the Instituto Nacional de Psiquiatría Ramón de la Fuente Muñiz (INPRFM, Mexico City, Mexico). They were managed in strict accordance to the specifications of current national legislation (Norma Oficial Mexicana: NOM-062-ZOO-1999) and the general principles of laboratory animal care (NIH publication #85-23, revised in 1985). Experimental procedures were approved by the institutional bioethics committee: Comité de Ética en Investigación del INPRFM, approval code IC122037.0 (16 July 2012). Mice were housed in a temperature-controlled (21–22 °C) room (eight per cage) under inverted light/dark conditions (12:12 h, lights on at 20:00 h (ZT0)). Animals had access to Purina^®^ (Brentwood, MO, USA) rodent chow and water ad libitum. All experimental procedures were carried out under dim red light.

### 4.2. Serum Melatonin Detection by DIESI-MS

Swiss Webster mice were sacrificed rapidly by decapitation at ZT12, 15, 18, 19, 21, and 0. The blood of each subject was collected and immediately centrifuged at 9000 rpm, during 15 min at 4 °C (Smart R17 refrigerated microcentrifuge, Hanil Science Industrial, Gimpo, Korea). Serum samples were protected from light and kept frozen (−20 °C) until analysis day.

DIESI-MS were conducted on Bruker MicrOTOF-QII system by an electrospray ionization (ESI) interface (Bruker Daltonics, Billerica, MA, USA) operating in the positive ion mode.

An aliquot (10 µL) of serum was resuspended in 1 mL of methanol, filtered through a 0.25 μm polytetrafluoroethylene (PTFE) filter and diluted 1:100 with methanol to avoid saturation of the capillary and cone soiling. To improve ionization, 25 μL of pure formic acid were added to 475 μL of diluted sample [final concentration 5% (*v*/*v*) formic acid]. Diluted and acidified samples were directly infused into the ESI source and analyzed in positive mode. A constant volumetric flow rate (8 μL/min) was achieved using a 74900-00-05 Cole Palmer syringe pump (Billerica, MA, USA). Capillary voltage was set to 4500 V, and nitrogen was used as a drying and nebulizing gas, using a flow rate of 4 L/min (0.4 Bar) with a gas temperature of 180 °C. Continuous spectra were collected in a *m*/*z* range of 50–3000, with total run duration of 1 min, a scan time of 10 s, and an interscan time of 0.1 s, producing six spectra per sample.

The mass spectrometer was operated at a resolution of 11,000 (FWHM) at mass 1622.0290 *m*/*z* in positive ion modes at a capillary voltage of 4500 V (positive). The spectrometer was calibrated with an ESI-TOF tuning mix calibrant (Sigma-Aldrich, Toluca, Estado de México, Mexico).

Finally, precursor ion scans (MS/MS) were performed using positive electrospray ionization (ESI-+) with appropriate mass set. According to the obtained pattern, suitable fragments were analyzed by a Bruker Compass Data Analysis 4.0 (Bruker Daltonics), which provided a list of possible elemental formulas using Generate Molecular Formula Editor, as well as a sophisticated comparison of the theoretical with the measured isotope pattern (σ value) for increased confidence in the suggested molecular formula (Bruker Daltonics Technical Note 008, 2004).

The accuracy threshold for confirmation of elemental compositions was established at 5 ppm.

### 4.3. Pharmacological Treatments

All drugs were intraperitoneally (i.p.) administered in a total volume of 10.0 mL/kg (body weight). Doses are expressed as milligrams per kilogram of mouse body weight. Melatonin (MEL) (Sigma-Aldrich Corp., St. Louis, MO, USA) was dissolved in the minimum amount of absolute ethanol and then in isotonic (0.9%) saline solution. Ethanol concentration in that solution was 0.006%, so the vehicle (VEH) tested had that same amount of ethanol. Fluoxetine (FLX) and imipramine (IMI) (Sigma-Aldrich Corp., St. Louis, MO, USA) were dissolved in isotonic saline solution. Experiment series comprised independent groups of 8 animals for each specified drug treatment and behavioral test.

Single administration at ZT11 was as follows: Either MEL or its VEH were administered at ZT11 (1 h before the lights were off), and behavioral tests were assessed 7.5 h later (ZT18.5).

Single administration at ZT18 was as follows: Drugs were administered at ZT18 (the middle of the dark phase), 30 min before the behavioral tests.

Triple scheme administration (for the FST only) was as follows: First administration of the drugs was at 24.5 h before the test (ZT18; immediately after the pre-test session); the second injection was applied one hour before lights were off (ZT11; 7.5 h before the test); finally, the third administration was at ZT18, 30 min before the test [39].

### 4.4. Behavioral Tests

#### 4.4.1. Forced Swimming Test (FST)

Mice were individually placed into glass cylinders (height: 21 cm, diameter: 14.5 cm) containing 15 cm of water at 23 ± 1 °C. All animals were forced to swim for 15 min (pre-test), followed by a 5-min session (test) 24 h later. The total immobility time was measured in seconds. The immobility behavior was considered when mice remain floating and treading just enough to keep the nose above the water. After the swimming sessions, mice were removed from the cylinder and carefully dried, placed in heated cages for 20 min and then returned to their home cages. All experimental sessions were videotaped and later analyzed [39,56,57].

#### 4.4.2. Tail Suspension Test (TST)

Acoustically and visually isolated mice were fastened by the tail and suspended 50 cm above the surface of a wooden box by adhesive tape placed approximately 1 cm from the tip of the tail. Each mouse was suspended for a total of 6 min and long-lasting immobility was recorded during the final 4 min of the test. Immobility behavior was scored only when the mouse remained passively hung and completely motionless [58,59].

#### 4.4.3. Open Field Test (OFT)

To discard unspecific effects, all experimental mice were subjected to the OFT. Motor activity was measured in an apparatus consisting of an opaque-plexiglas box (40 × 30 × 20 cm), which surface was divided into 12 equal squares (11 × 11 cm). The animal was placed in a corner of the cage, and its behavior was videotaped over 5 min. An observer, who was blind to the pharmacological treatments, registered the total number of times (counts) that the animal crossed a square during the test. A count was considered when the animal totally crossed from one square to the next. Any change in the number of counts is considered as an alteration in the locomotor activity. The test box was carefully cleaned after each session. To prevent behavioral changes of the animals after the first experience, mice were tested only once [60].

### 4.5. Statistical Analysis

Comparisons between the two experimental groups were assessed with the Student’s *t*-test. Data that met criteria of normality (Kolmogorov–Smirnov test) and variance equality were analyzed using analysis of variance (ANOVA). Bonferroni’s test for multiple comparisons versus control group was applied when the ANOVA showed significant difference; *p*-values ≤ 0.05 were statistically significant (*p* * ≤ 0.05, *p* ** ≤ 0.01, and *p* *** ≤ 0.001).

All statistical analysis and graphics were carried out using the Sigma Plot software version 12.0 (Systat software, San Jose, CA, USA).

## Figures and Tables

**Table 1:** Mice ambulatory activity assessed through the Open Field Test (OFT).

Treatment (mg/kg)	Count Number/5 min	Rearing Number/5 min
VEH	43.87 ± 4.42	34.50 ± 3.95
MEL 4	41.12 ± 2.48	25.37 ± 2.18
MEL 16	38.50 ± 3.74	25.12 ± 2.36
	F_(2,23)_ = 0.54, *p* = 0.58	F_(2,23)_ = 3.29, *p* = 0.05
VEH	43.87 ± 4.42	34.50 ± 3.95
FLX 10	36.00 ± 3.576	25.62 ± 3.17
FLX 15	47.75 ± 2.66	28.37 ± 0.75
	F_(2,23)_ = 2.72, *p*= 0.08	F_(2,23)_ = 3.06, *p* = 0.10
VEH	43.87 ± 4.42	34.50 ± 3.95
IMI 12.5	36.12 ± 3.52	20.87 ± 3.45
IMI 25	41.75 ± 5.89	34.50 ± 7.72
	F_(2,23)_ = 1.48, *p* = 0.25	F_(2,23)_ = 2.69, *p* = 0.09

## Abbreviations

CSF	Cerebrospinal fluid
DIESI/MS	Direct Infusion Electrospray Ionization Mass Spectrometry
FST	Forced Swimming Test
OFT	Open Field Test
SSRIs	Selective Serotonin Reuptake Inhibitors
TST	Tail Suspension Test
ZT	Zeitgeber time
